# Growth Characteristics of Electro-Water Mixed Branches in Acid-Base Solution Based on Frequency Dielectric Spectroscopy Analysis

**DOI:** 10.3390/polym18091092

**Published:** 2026-04-30

**Authors:** Songwei Li, Bo Zhu, Xinyu Zhang, Bo Yang

**Affiliations:** School of Electrical and Electronic Engineering, Harbin University of Science and Technology, Harbin 150080, China; 2303010807@stu.hrbust.edu.cn (S.L.); 2320310260@stu.hrbust.edu.cn (X.Z.); 2320310165@stu.hrbust.edu.cn (B.Y.)

**Keywords:** cross-linked polyethylene cable, mixed branches, growth characteristics, frequency dielectric spectroscopy

## Abstract

In order to explore the effect of pH value of the solution on the growth characteristics of electro-hydro mixed branches of cross-linked polyethylene (XLPE) cables, an electro-hydro mixed branch experimental platform with different pH values was built to accelerate the aging of XLPE cables. The growth characteristics of electro-hydro mixed branches under different pH environments were systematically observed and analyzed by combining macroscopic dielectric properties test with microscopic morphology detection. The macroscopic test results show that the aging degree of the cable is more serious in the acidic or alkaline environment. When there are electrical tree defects in the insulation, acidic or alkaline solutions with different pH values will promote the accelerated aging of mixed branches, and the acceleration effect of acidic environment is more significant. After microscopic detection of sample slices with different acidity and alkalinity, it was found that both acidic and alkaline environments could accelerate the growth of mixed branches. On the basis of electrical trees, the strong acid and strong alkali environment was more suitable for the development of mixed branches than the weak acid and weak alkali environment, and the promotion effect of acidic solution was more prominent. At the same time, this study also deeply analyzed the conversion mechanism of electrical tree to water tree in cables under different pH conditions. Finally, through the correlation analysis between the dielectric performance parameters and the branch density of different groups of samples, the fitting model of the branch density on the macroscopic dielectric performance parameters is obtained by curve fitting, which provides an effective non-destructive testing method for cable multi-branch aging. These results reflect the structure–property relationship of XLPE polymer under acid-base corrosion and electric field coupling and reveal the microstructure degradation mechanism of polyethylene insulation.

## 1. Introduction

Cross-linked polyethylene (XLPE) cables are widely used in power transmission and distribution networks due to their excellent electrical insulation performance and mechanical stability [1]. However, in the manufacturing process, the insulating layer is prone to produce primary defects such as microcracks and holes due to technical limitations. In the subsequent laying and operation process, the mechanical stress will further aggravate the development of these defects [2]. After water invades the insulation layer through a variety of ways, it will induce the growth of water trees under the action of high electric field. Some water trees will be converted into electrical trees under overvoltage conditions, causing electric field distortion, which may eventually lead to cable insulation breakdown and seriously threaten the safe operation of the power grid [3]. Traditional research mostly regards electrical trees and water trees as independent aging phenomena, but the actual cable operating environment is complex. Electro-hydro mixed branches are composite defects formed by the synergistic growth and mutual conversion of electrical trees and water trees under the combined action of electric field, water, and acid–base environment. Unlike pure electrical trees caused by discharge or pure water trees caused by water erosion, mixed branches contain both discharge channels and microporous structures, leading to fundamentally different aging behavior. The discontinuous action of overvoltage and the continuous erosion of water will lead to the synergistic occurrence and mutual influence of two types of branch defects, and the degradation mechanism is significantly different from that of single branch defects [4]. Studies have shown that water trees and electrical trees do not develop in isolation, but coexist in the insulating layer and may be converted to each other. For example, the front end of the water tree can be connected to the electrical tree to form a composite defect [5]. At present, scholars have carried out preliminary exploration on the initiation and growth mechanism of XLPE multi-branch aging. Jiang et al. have found that wet water trees will hinder the growth of electrical trees, while dry water trees will accelerate the deterioration of electrical trees. The constraint of water molecules on high-energy particles is a key factor affecting the development of electrical tree defects [6]. Chen Junhao et al. have shown that under the combined action of overvoltage and overheating conditions, the tip of the open water tree branch can easily induce electrical tree branches, which can lead to insulation breakdown in severe cases and pose a direct threat to the safe and stable operation of the cable [7]. Li Tianhua et al. analyzed the mechanism and characteristics of the transformation from electrical tree to water tree by means of finite element electric field simulation and combined with the actual operating conditions of the cable [8]. Dong Yunzhi and others have carried out further research on the transformation characteristics of water tree branches to electrical tree branches. The results show that the degree of cross-linking will have a certain impact on the transformation process [9]. In addition, the influence of environmental factors on branch aging has also received extensive attention. Hayashi Y et al. found that temperature has a significant regulatory effect on the process of water trees triggering electrical trees, and a high temperature environment will accelerate the degradation of water trees and increase their growth rate [10]. Through the electrical tree initiation experiment under AC voltage, Zhou Kai et al. constructed a two-dimensional simulation model of electrical tree and water tree and proposed the possible mechanism of the transformation of electrical tree to water tree in service-aged XLPE cable [11]. Zhang Chunshuo et al. analyzed the influence of different acid and alkali environments on the growth characteristics of XLPE cable water tree from the perspective of ion characteristics, which laid a foundation for the subsequent research on acid and alkali [12]. Although some progress has been made in the existing research, systematic research on the mixed growth of electrical tree and water tree is still scarce. The key issues, such as the influence mechanism of cable operating environment (especially solution pH) on the aging of mixed trees and the evaluation of insulation life, have not been fully solved. Scholars’ explanation of the mechanism of dendritic defects is still not perfect. The existing conclusions are mostly based on the electric field simulation model, and the influence of historical dendritic structure and channel derivatives on insulation aging is not fully considered [13]. In different acid-base environments, the non-synchronous development law of mixed branches and the evolution mechanism of defect characteristics during aging still need to be further explored [14].

Compared with existing studies that mainly focus on the aging behavior of individual electrical trees or water trees, this study concentrates on the synergistic growth of electro-hydro mixed branches in XLPE cables under different pH environments. This condition is more consistent with the actual service environment where water, electric field, and acid-base effects coexist. The growth characteristics of mixed branches, the influence of pH, and the conversion mechanism between electrical trees and water trees have rarely been systematically studied. This work supplements the aging mechanism of XLPE insulation under acid-base environments and provides a reference for non-destructive condition evaluation of cables.

In order to study the influence of different pH values on the aging growth characteristics of XLPE cable electro-hydro mixed branches, a cable electro-hydro mixed branch aging platform that can change the pH value of the solution was built. Firstly, the needle electrode was used to accelerate the electrical tree aging of XLPE cable samples, and then the water needle method was used to accelerate the water tree aging. The complex dielectric constant, dielectric loss factor, and AC conductivity at different frequencies were measured by frequency domain dielectric spectroscopy (FDS), and the polarization relaxation and conductivity characteristics of insulating medium were studied. The morphology of the branches in the sample was observed by optical microscope and photographed. The pore morphology of dendritic defects was observed by scanning electron microscopy, and the area of mixed dendritic micropores was measured. The samples were measured by Fourier infrared spectrometer before and after aging, so as to detect the changes in aging degree and microstructure of the samples. Finally, by combining macro parameters and micro parameters, the cable samples are analyzed for the aging growth characteristics of electro-hydraulic mixed branches and cable aging assessment. Acid-base pollution conditions. This study focuses on the polymer chain fracture, microstructure evolution, and oxidative degradation of XLPE, revealing the intrinsic relationship between polymer structure damage and macroscopic dielectric aging.

## 2. Experimental Design and Measurement Method

### 2.1. XLPE Cable Sample Preparation

The cable sample needs to be pretreated before the cable electrical tree-water tree joint aging test. The grouping of electro-water mixed branch aging test under different pH values is shown in Table 1.

In Table 1, the number before “-” is the pH value of the aging solution, and the number after “-” is the aging time. The number 1 represents the aging time of 21 days, and the number 2 represents the aging time of 42 days. In this paper, YJV22-8.7/15 kV cross-linked polyethylene (XLPE) cable is selected as the test object. The specific pretreatment and electrical tree aging test process is as follows:

First, the outer sheath, armor layer, and filler of the three-phase cable are completely peeled off, and the single-phase cable is processed into a short sample of 50 cm per section by means of a cutting machine. After cleaning the surface of the outer semi-conductive layer of the sample, the insulating layer of 2 cm at both ends was removed to expose the copper core, and a copper nose was installed at one end of the copper core to facilitate subsequent wiring. The outer semi-conductive layer at 5 cm away from the conductor at both ends of the sample is stripped to expose the cross-linked polyethylene layer, so as to avoid the surface discharge caused by moisture in this area during the aging test. At the same time, the exposed cross-linked polyethylene layer is set with waterproof heat-shrinkable pipe, which is heated by BK-414001 hot air gun, so that the heat-shrinkable pipe is closely attached to the cross-linked polyethylene layer to play a moisture-proof role. After the surface of the sample to be pretreated is cleaned with anhydrous ethanol, the electric stress evacuation adhesive is pasted at both ends of the semi-conductive layer. In the semi-conductive layer area, a steel needle electrode with a curvature radius of 3.0 ± 0.2 μm and a diameter of 1 mm is inserted perpendicular to the cable core axis at an interval of 1 cm, and the insertion depth is 3 mm. Subsequently, the cable sample was immersed in No. 45 transformer oil, the electrode was connected and a 50 Hz AC voltage was applied, and the voltage was gradually increased to 12 kV according to the 1 kV gradient. In the process of electrical tree aging test, each steel needle electrode needs to be separately pressurized to ensure uniform electrical stress distribution.

After the test, the electrical stress relief adhesive and electrode on the surface of the cable sample were removed, and the residual transformer oil was washed with anhydrous ethanol. A 40 mm heat-shrinkable tube was set in the semi-conductive layer in the middle of the sample, and both ends of the heat-shrinkable tube were sealed by a hot air gun (keeping the middle area unchanged). The middle cavity was used to store the solution, and an opening of about 10 mm was reserved in the middle of the heat-shrinkable tube for subsequent electrode placement and solution injection.

### 2.2. XLPE Cable Electro-Hydraulic Mixed Branch Aging Experiment

**Figure 1:**
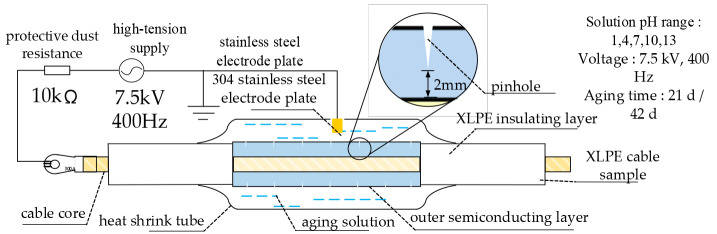
Schematic diagram of electro-hydraulic mixed branch aging platform.

After the pretreatment of XLPE cable samples is completed, the electro-hydraulic mixed tree aging experiment is carried out. The mixed tree aging experiment platform under different pH conditions is shown in Figure 1. During the experiment, a sinusoidal AC voltage with an effective value of 7.5 kV and a frequency of 400 Hz is applied to the sample. According to the schematic diagram, the cable sample is connected with the power supply by high-voltage wire to form a closed loop. The 10 kΩ resistor in series in the circuit is a protection resistor, which limits the short-circuit current and impulse current to avoid damage to the equipment due to excessive current. In order to prevent partial discharge between the sample and the ground during the test, it is necessary to arrange the cable sample overhead and control the height from the ground at about 20 cm. In this experiment, 304 stainless steel electrode was selected. The electrode was fixed by bracket, and the height could be adjusted flexibly. During installation, it is necessary to ensure that the electrode is in full contact with the aging solution and, at the same time, avoid touching the outer semi-conductive layer of the cable to ensure the safety and data accuracy of the experiment.

### 2.3. XLPE Cable Aging Measurement Method

In this paper, the effects of different pH environments on the aging and growth characteristics of XLPE cable electro-hydro hybrid trees are studied. The specific schemes and analysis methods are as follows: In the experiment, solutions with pH values of 1, 4, 7, 10, and 13 were selected to construct different acid-base environments, and XLPE cables were subjected to electro-hydraulic mixed branch aging treatment. During the experiment, all kinds of data were recorded in detail, and in-depth analysis was carried out to ensure the accuracy and reliability of the research results. The analysis adopts a multidimensional characterization method combining macro and micro: At the macro level, the cable aging degree is evaluated by FDS measurement; at the micro level, optical microscopy, scanning electron microscopy, and infrared spectroscopy were used to obtain key information, such as dendritic aging morphology, section characteristics, elemental composition, and chemical bonds. The development and evolution of defects in different acid-base environments are analyzed by data comparison. Based on the related theories of electrophysics, electrochemistry, and polymer materials, the mechanism of different acid-base environments on the dendritic aging of XLPE cables is systematically explained.

The pH values of 1, 4, 7, 10, and 13 were selected to construct typical strong acid, weak acid, neutral, weak alkaline, and strong alkaline environments, respectively. This grouping can clearly reveal the influence of acid-base strength and ion concentration on the growth of electro-hydro mixed branches. The pH = 1 and pH = 13 represent strong acid and strong alkali environments, which are used to analyze the severe erosion effect. The pH = 4 and pH = 10 represent weak acid and weak alkali environments, which are close to mild acidic/alkaline pollution in actual cable trenches. The pH = 7 is the neutral environment as a reference group. This setting enables a systematic comparison of the growth characteristics, microscopic morphology, and dielectric response of mixed branches under different pH conditions, ensuring the experimental results are comprehensive and comparable.

## 3. Macroscopic Parameter Test (FDS) of XLPE Cable

In this paper, FDS test is used as the main macro-dielectric performance test to detect the insulation performance of cable samples. The insulation performance of cable samples was tested by Megger IDAX300 broadband dielectric response analyzer from Dover Megger Group Co., Ltd. Based on the three-electrode method, the cable core is connected to the high-voltage electrode, the semi-conductive layer copper foil is used as the measuring electrode, and the XLPE surface is pasted with a copper foil shielding ring to ground. The test frequency range is 0.01~1000 Hz, the excitation voltage is 1 kV, the ambient temperature is 20 °C, and the complex permittivity and dielectric loss factor curves are collected.

The calculation method of the geometric capacitance *C*_0_ of the cable sample is

(1) $$C_{0}=\frac{2\pi \varepsilon_{0}L}{\ln(\frac{R_{2}}{R_{1}})}$$

In the formula, *L* is the length of cable insulation layer, m, and *R*_1_ and *R*_2_ are the inner diameter and outer diameter of the insulating layer, respectively, m.

The DC conductivity expression of the cable is

(2) $$\sigma_{0}=\frac{\varepsilon_{0}}{C_{0}U_{0}}[i_{\mathrm{pol}}(t)-i_{\mathrm{depol}}(t)]$$

From the above equation, the frequency domain relationship of dielectric loss factor of XLPE cable insulation can be obtained:

(3) $$\tan\delta (\omega )=\frac{\varepsilon^{\prime \prime }(\omega )}{\varepsilon^{\prime }(\omega )}=\frac{\frac{\sigma_{0}}{\varepsilon_{0}\omega }}{\varepsilon_{\infty }+\chi^{\prime }(\omega )}+\frac{\chi^{\prime \prime }(\omega )}{\varepsilon_{\infty }+\chi^{\prime }(\omega )}$$

In the formula, tan*δ*(ω) is the frequency domain dielectric loss factor of the cable, and *ε*′(ω) and *ε*″(ω) are the real and imaginary parts of the complex permittivity, respectively.

The nonlinear characteristic parameter λ is introduced to quantitatively characterize the aging degree, and the calculation formula is as follows [15]:

(4) $$\lambda_{i}=\frac{S_{i}U_{i}-S_{i+1}U_{i+1}}{\sqrt{U_{1}^{2}+U_{2}^{2}+\cdots +U_{i}^{2}+U_{i+1}^{2}}}$$

(5) $$\lambda =\frac{\lambda_{1}+\lambda_{2}+\cdots +\lambda_{i}}{i}$$

*S_i_* is the integral value of the tan*δ*-*f* curve corresponding to the voltage *U_i_*.

**Figure 2:**
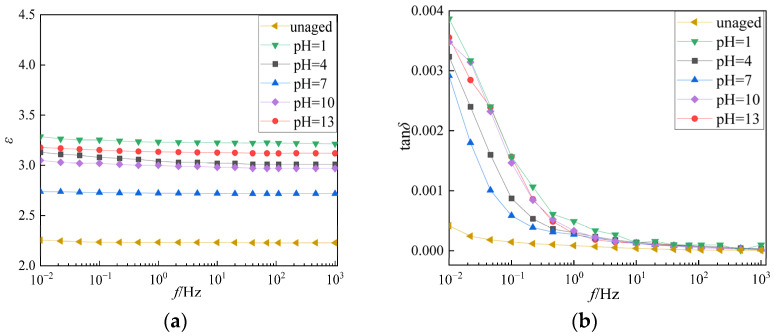
The *ε*-*f* curve and tan*δ*-*f* curve of the samples aged for 42 days in different pH solutions. (**a**) The *ε*-*f* curve of 42 days aging sample; (**b**) The tan*δ*-*f* curve of aged 42 days sample.

According to the test grouping settings in Table 1, the XLPE cables after electrical tree aging were placed in the environment of pH = 1, pH = 4, pH = 7, pH = 10, and pH = 13 for mixed branch joint aging. After aging, the cables were subjected to FDS test. The FDS measurement results of the aged 42 days samples at a frequency of 0.01–1000 Hz are shown in Figure 2.

It can be seen from Figure 2 that the FDS test results show that when the solution pH = 1 and pH = 13, the dielectric loss factor of the sample at low frequency is larger, followed by the weak acid and weak alkali environment, and the dielectric loss of the cable after aging in the neutral environment is the smallest.

**Figure 3:**
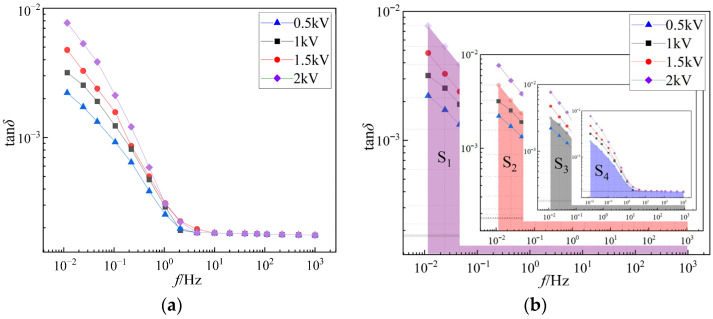
The tan*δ*-*f* curve and its integral diagram of the sample pH4-2. (**a**) The tan*δ*-*f* curve of the sample pH4-2. (**b**) The integral diagram of tan*δ*-*f* curve of sample pH4-2.

In addition, the aging degree of cable after mixed branch aging was further quantitatively judged by measuring the FDS spectra of aging samples with different concentrations under applied voltages of 0.5 kV, 1 kV, 1.5 kV, and 2 kV. In order to ensure the accuracy of measurement, each sample was measured three times at room temperature and atmospheric pressure. The tan*δ*-*f* curve and its integral diagram of the sample pH4-2 are shown in Figure 3.

From Figure 2, it can be seen that the *ε* is the smallest in the neutral environment, the *ε* is higher in the acidic and alkaline environments, and the farther the pH deviates from the neutral, the larger the *ε*, among which the pH = 1 and pH = 13 increase most obviously. Under the same deviation degree, the *ε* in the acidic environment is slightly higher than that in the alkaline environment, indicating that the acid-base environment promotes the growth of mixed branches, and the effect of H^+^ is stronger than that of OH^−^. tan*δ* increases significantly with the decrease of frequency, and the increase in low-frequency band is obvious. The tan*δ* in the neutral environment is the smallest in the whole frequency range, and the acid-base environment increases the tan*δ*. The low-frequency loss is the largest at pH = 1 and pH = 13. The tan*δ* in the acidic environment is generally higher than that in the corresponding alkaline environment, indicating that the acid-base accelerated aging, and the acid-accelerated effect is more prominent.

It can be seen from Figure 3 that as the excitation voltage increases from 0.5 kV to 2 kV, tan*δ* increases in the full frequency band, and the increase in the low frequency band is more significant, reflecting the nonlinear dielectric properties of aging insulation. High voltage will aggravate ion migration and polarization loss, which is related to the defect path formed by mixed branches. The integral area S reflects the total dielectric loss of insulation under different voltages. The higher the voltage, the larger the integral area and the more serious the total loss. Combined with the calculation of the nonlinear characteristic parameter *λ*, *λ* can be used to identify and analyze the aging degree, defect condition, and insulation performance of the cable. The value of *λ* is positively correlated with the aging degree of XLPE cable, that is, the larger the value of *λ*, the more serious the aging of the cable’s electro-hydro mixed branches, and the more significant the deterioration of insulation performance, which provides a quantitative basis for comparing with the aging degree of other pH samples.

Although the absolute change of tan*δ* is not prominent under different excitation voltages, the stable and consistent increasing trend in the full frequency band, especially the more obvious growth in the low-frequency region, can still effectively indicate the nonlinear dielectric characteristics of aged insulation. This trend originates from the enhanced ion migration and interfacial polarization in the microporous channels of mixed branches under higher electric field strength, which is consistent with the aging mechanism of electro-hydro mixed branches.

According to the FDS test results, the nonlinear characteristic parameter *λ* of the samples with different pH values was calculated. The calculation results are shown in Table 2 and Table 3.

The average value of the *λ* value of the unaged cable is calculated to be 132.3. The nonlinear characteristic parameters can characterize the polarization loss and conduction loss of the cable sample, which are positively correlated with the voltage and the aging degree of the cable. Under the aging cycle of 21 days, the *λ* values of the samples treated with strong acidic (pH = 1) and strong alkaline (pH = 13) solutions were significantly higher than those of the neutral samples and were much higher than the *λ* reference values of the unaged cables. The results show that the acidic or alkaline environment can significantly promote the aging process of XLPE cable electro-hydro mixed tree and enhance the nonlinear dielectric properties of cable insulation. The neutral environment is the slowest acid-base environment for cable insulation degradation, which is consistent with the conclusion that the dielectric loss of neutral environment is the smallest in the macroscopic dielectric property test. Although the *λ* value of pH7-2 is significantly higher than that of pH7-1, it is not the neutral environment itself that accelerates the aging, but the cable insulation in the neutral environment will still undergo basic aging under the combined action of water and alternating electric field, and the long period of 42 days makes this natural aging effect without chemical action continue to accumulate. At the same time, the electrical tree defects formed by the pre-aging of the sample will slowly transform to the water tree over time and finally reflect the jump of the *λ* value, but its value is still not more than the strong acid sample of the same period. In the same aging cycle, the *λ* values of strong acid (pH = 1) and strong alkali (pH = 13) samples were significantly higher than those of weak acid (pH = 4) and weak alkali (pH = 10) samples, indicating that the higher the concentration of H^+^ and OH^−^ in the solution, the more obvious the acceleration effect on the aging of cable mixed branches. Under the same alkalinity and acidity, the λ value of the acidic sample is higher than that of the alkaline sample, indicating that the acidic environment has a more significant effect on the nonlinear deterioration of cable insulation than the alkaline environment of the same grade, reflecting that the damage effect of H^+^ on XLPE molecular chain is stronger than that of OH^−^. Moreover, under the same acidity and alkalinity conditions, the *λ* values of the samples after 42 days aging are higher than those of the 21 days aging samples, indicating that as the aging time increases, the aging effect of the acid-base environment on the cable continues to accumulate, and the polarization loss and conductivity loss of the cable insulation continue to increase. The nonlinear dielectric properties are further enhanced, and the growth and development of mixed branches become more serious over time. There is an order of magnitude difference between the *λ* value of the unaged cable and the aged sample, and the *λ* value of the aged sample shows regular changes with the acid-base strength and aging time, which is highly consistent with the growth trend of the cable mixed branch. This shows that the nonlinear characteristic parameter *λ* can effectively reflect the aging degree of XLPE cable electro-hydro hybrid tree. The larger the value of *λ*, the more serious the polarization loss and conductivity loss of cable insulation, and the more significant the aging of hybrid tree.

## 4. Micro Parameter Test of XLPE Cable

After the frequency domain dielectric spectroscopy (FDS) test of XLPE cable is completed, the pinhole position of the semi-conductive layer is sliced and prepared. The specific processing flow is as follows: firstly, the aging area of the cable sample is cut into 5 cm long segments, and the internal residual cable core is removed without damaging the hole structure. Subsequently, the slicer was used to slice at each pinhole, and the thickness of the single slice was controlled to be 100 μm. Due to the random error in the slicing process, 20 complete slices were selected for each sample as observation samples. The above observation samples were divided into three groups on average and treated differently. The first group was stained with 3% methylene blue solution for observing the microstructure of mixed branches. The second group was treated with liquid nitrogen low-temperature brittle fracture along the cross section of mixed branches for scanning electron microscopy (SEM) observation. The third group was directly used for Fourier transform infrared spectroscopy (FTIR) analysis without additional treatment.

### 4.1. Optical Microscope Observation

In order to observe the morphology of mixed branches in XLPE cables, it is necessary to dye the cable slice samples first and then observe them with optical microscope. The specific operations are as follows: The prepared slices are placed in methylene blue dye and placed in an ultrasonic heating box. The water bath is heated to 90 °C, and the residual dye on the surface is washed after the slices are fully dyed; the stained sections were placed under an optical microscope and focused until the morphology of the branches was clear and distinguishable. The morphology was photographed and archived, and the length and width parameters of the mixed branches were marked in the imaging map. In order to improve the observation clarity and accuracy of the branch morphology, the captured images are converted into the film format.

The optical microscope used in this experiment is Keyence VH-Z100R from Keyence Corporation, Osaka, Japan and rectangular XSP-8C from Shanghai Diyilun Optical Instrument Co., Ltd., Shanghai, China. The supporting imaging camera equipment is VHX-500FE from Keyence Corporation, Osaka, Japan.

**Figure 4:**
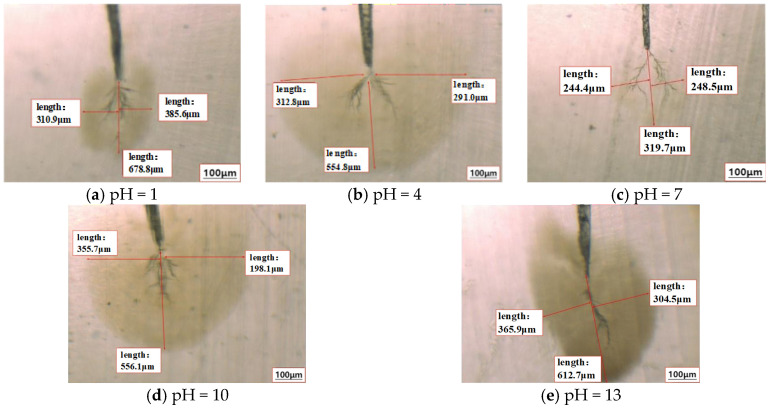
The microstructure of the samples aged for 21 days in different acidity and alkalinity test groups.

The cable samples after the macroscopic test were sliced and divided into three groups. The dendritic defect area in the first group of flake samples was observed by optical microscope, and the dendritic morphology of the cable samples after 21 days of mixed tree aging under different pH solution environments was shown in Figure 4.

It can be seen from Figure 4 that when the aging solution is neutral, the length of the mixed branch formed by the sample after 21 days of aging is relatively short, but the degree of staining in the defect area is deeper. This indicates that the length and diameter of the mixed branches are affected by the concentration of H^+^ and OH^−^ in the solution, and the presence of the two ions will promote the development of the mixed branches to a longer and finer morphology.

Considering that the dendritic defects actually generated in XLPE cables are three-dimensional structures, if the slice observation is only carried out in the direction perpendicular to the cable section, it is easy to introduce large errors due to the incomplete dendritic morphology. In order to quantitatively characterize the development degree of branch defects more accurately, this paper introduces branch volume as an evaluation index. By measuring the transverse and longitudinal dimensions of water tree defects, the mixed branch area is approximately equivalent to an ellipsoid, and the growth degree of mixed branches is quantitatively described by ellipsoid volume. Since the electrical tree branches are usually wrapped in the central region by water trees, the volume calculation formula corresponding to the mixed tree branch model is as follows:

(6) $$V=\frac{4\pi }{3}L_{1}^{2}L_{2}-\frac{3\pi }{4}\sum_{i=1}^{n}d_{i}^{2}l_{i}-\frac{3\pi }{4}hL_{3}^{2}$$

For Equation (6), the second term on the right side of the equation is the approximate calculation result of the volume of the electrical tree in the water tree wrapping area, and the third term corresponds to the volume of the pinhole structure under the water tree wrapping. When the water tree area shows obvious semi-circular characteristics, the corresponding calculation formula can be simplified as shown in Equation (7):

(7) $$V=\frac{2\pi }{3}L_{1}^{2}L_{2}-\frac{3\pi }{4}\sum_{i=1}^{n}d_{i}^{2}l_{i}$$

In the formula, *V* is the approximate volume of mixed branch defects, mm^3^; *L*_1_ is the maximum transverse width of the mixed branch, mm; *L*_2_ is the length of the mixed branch from the initiation to the end, mm; *L*_3_ is the maximum diameter of the pinhole covered by the mixed branch area, mm; *h* is the length of the pinhole covered by the mixed branch area, mm; *d_i_* is the diameter of the ith electrical tree branch, mm; and *l_i_* is the distance from the ith electrical tree branch to the tip to the end of the branch, mm.

**Figure 5:**
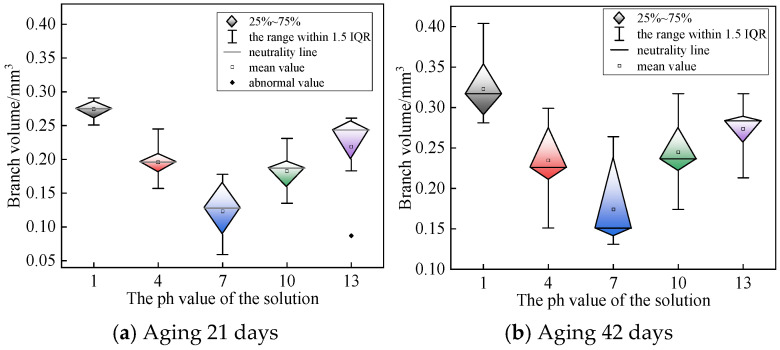
Branch volume of samples in different pH test groups.

Microscopic observation and marking were carried out on the specimens of the test group under different pH conditions, and the size of branch defects of each specimen was recorded one by one. The results are shown in Figure 5. It can be seen from Figure 5 that the mixed branch volume of the sample in acidic and alkaline environments is larger as a whole. For the samples aged for 21 days, acidic or alkaline solution had a significant promoting effect on the growth of mixed branches. At 42 days of aging, the difference in branch volume between different pH test groups was significantly reduced. This indicates that in the later stage of aging, the promoting effect of electrical trees on the growth of water trees is gradually weakened, and the growth rate of water trees is mainly controlled by the diffusion rate of the solution inside the material.

From the comparison results of Figure 4 and Figure 5, it can be seen that the radius of Na^+^ ion is much larger than that of H^+^. Although H^+^ usually exists in the form of H_3_O^+^ in aqueous solution, it has a very high migration rate after obtaining energy under the action of AC electric field. The high-speed moving H^+^ continuously impacts the cross-linked polyethylene (XLPE) molecular chain, which eventually leads to the fatigue fracture of the molecular chain. Under the alternating electric field, H^+^ ions present as hydronium ions H_3_O^+^ in aqueous solution have small ionic radius, light mass, and high migration velocity. Driven by the periodic electric field force, high-speed H^+^ repeatedly impacts and collides with XLPE molecular chains. The long-term cumulative impact causes continuous fatigue damage to the C–C bonds and C–H bonds on the molecular chains. With the increase of impact times and aging time, the molecular bonds gradually break, resulting in the fatigue fracture of XLPE molecular chains, which further promotes the formation and propagation of microcracks and micropores, and accelerates the growth of electro-hydro mixed branches. Due to the small radius and light weight of H^+^ ions, the impact behavior of H^+^ ions on the molecular chain has strong randomness and dispersion. Therefore, the diameter of the mixed branch holes and cracks formed is relatively small, resulting in the dyeing effect of methylene blue dye is not significant enough. In contrast, the mobility of Na^+^ is significantly lower. Because of its large ion radius and mass, the impact of Na^+^ on the molecular chain is more concentrated. Under the action of external electric field, the migration rate is slow, and the impact energy is more likely to accumulate locally. The final mixed branch length is shorter, but the hole diameter is larger, so the dyeing effect is deeper.

The experimental results further show that the growth rate of mixed branches is closely related to the type and concentration of ions in the solution. In the Na^+^-dominated system, although the length of the mixed branch is shorter, the cracks and holes formed by ion impact are more significant, which makes the dye more sensitive to the defect area. In the alkaline environment, there are a lot of OH^−^ in the solution. Although the ionic radius of OH^−^ (137 pm) is smaller than that of Cl^−^ (181 pm), it has a unique migration mechanism. Under the applied AC electric field, the movement rate of OH^−^ is relatively faster, which can also cause the molecular chain to break and eventually form a mixed branch, and its mechanism of action is similar to that of H^+^. In the weak acid or weak alkali solution, due to the low content of HCl and NaOH, the main component NaCl in the aging solution actually plays a decisive role in the aging process of the mixed branches. The development of mixed branches reflects the structural degradation of XLPE polymer under continuous ion impact and electric field fatigue.

### 4.2. SEM Observation

After the electrical-water mixed tree aging of XLPE cable, the aging area will form micro-channels and microporous structures. The micropore diameter of the mixed branch is usually in the range of 0.1 nm~5 μm, and the channel diameter is about 10 nm~100 nm, which is similar to the water tree defect scale. The diameter of the electrical tree channel is generally about 10 μm. In this paper, the pore morphology of dendritic defects was observed by scanning electron microscope. The scanning electron microscope is SU820 from Hitachi High-Tech Corporation, Tokyo, Japan. The diameter and area of the mixed dendritic pores were measured, and the types of dendritic channels were distinguished according to the pore diameter. The specific process of observing the dendritic defects of the cable by SEM is as follows: the aged sample slice is placed in liquid nitrogen for low-temperature brittle fracture, and the section is obtained in the aging area and sprayed with gold; taking the cross section of the branch channel as the observation object, the micropores of all the sample sections were observed, marked, and imaged. The micropore area of each group of samples was counted, and the influence of related factors on the pore characteristics was analyzed.

The equipment used in the experiment is Hitachi SU820 high-resolution field emission scanning electron microscope from Hitachi High-Tech Corporation, Tokyo, Japan. The resolution of the equipment can reach 1.3 nm at 1 kV acceleration voltage, and the magnification range is 20~800,000 times, which can clearly characterize the microstructure and fine structure of the sample surface.

**Figure 6:**
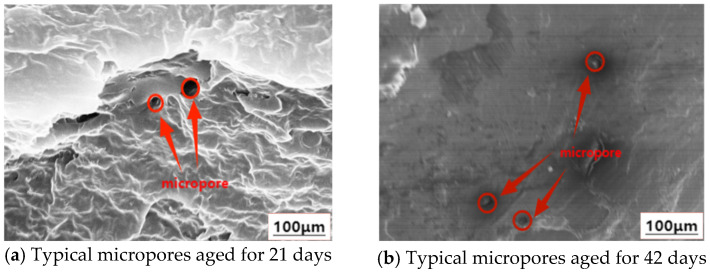
The morphology of typical micropores was observed by SEM.

The SEM microscopic observation of the mixed branch structure was carried out by using the above test method for the second group of sliced samples. After the cable specimen was sliced in the mixed branch defect area and subjected to low-temperature brittle fracture treatment in liquid nitrogen, the microscopic pores formed after the dendritic channel was cut off can be observed by scanning electron microscope. ImageJ software version 1.54r was used to label and calculate the area of the observed typical micropore images. The SEM morphology of the typical micropores was shown in Figure 6.

**Figure 7:**
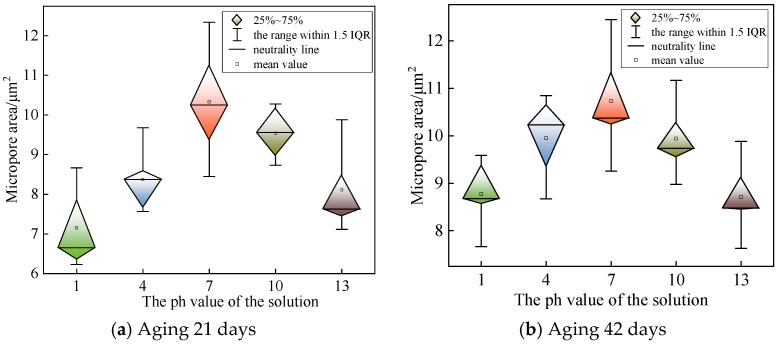
Micropore area of samples in different pH test groups.

The shape of the micropores is easily affected by the cross-sectional stress during the brittle fracture process, so that the observed micropores are mostly irregular in geometry, and the pore area is mostly in the range of tens to tens of square microns. According to the SEM observation results of each group of samples, 10 micropores with relatively regular morphology were selected for area statistics. The micropore area data of each group of samples were calculated and sorted out, and the box plot was drawn for comparative analysis. The statistical results of micropore area are shown in Figure 7.

From Figure 7, it can be seen that the H^+^, OH^−^, Na^+^, and Cl^−^ in the solution will have an impact on the XLPE molecular chain under the electric field force. Under the same external electric field conditions, different ions have different impact effects on the molecular chain due to their differences in radius, mass, and other characteristics. The radius of Na^+^ is about 10 times that of H^+^, and H^+^ has a higher migration rate in aqueous solution. There is a large amount of H^+^ in the strong acid solution, which mainly exists in the form of H^+^. Due to the small mass and high mobility of H^+^, its impact on the molecular chain is more dispersed, and the resulting mixed branch pores are finer. In the strong alkali solution, OH^−^ has a similar migration mechanism to H^+^, so the mixed branch growth degree of the sample in the alkaline environment is similar to that in the acidic environment. In the alkaline environment, the cation is still Na^+^, and the anion changes from Cl^−^ to OH^−^. Because the radius of OH^−^ is smaller than that of Cl^−^, the micropore size formed after the destruction of XLPE molecular chain is smaller. At the same time, the migration rate of OH^−^ is about 2.5 times that of Cl^−^, which makes the generated mixed branch channel thinner and longer, showing that the branch volume is larger and the dyeing degree is lighter. The effects of acidic and alkaline solutions on mixed branches are common. Both of them can increase the volume of mixed branches and reduce the area of micropores.

### 4.3. Infrared Spectroscopic Analysis

Fourier transform infrared spectrometer (FTIR) is an important characterization method for analyzing the microscopic composition and molecular structure of substances. Its working principle is based on the characteristic absorption of infrared light by different chemical groups. By irradiating the sample with infrared radiation and recording the absorption signals at different wavelengths, the spectral information related to the type, content, and structure of chemical bonds in the sample can be obtained. Under the combined action of external electric field and water, XLPE insulation materials are prone to oxidative degradation reactions, resulting in the breakage of the main chain of the polymer, and the number and type of hydrophilic groups inside the material change accordingly. In the FTIR test, the absorption intensity of infrared light at different wavenumbers directly reflects the content of the corresponding chemical group and its interaction with infrared light. By comparing the infrared spectrum curves of the samples before and after aging, the position, strength, and peak shape of the characteristic absorption peaks can be clearly observed. These changes can directly reflect the evolution process of XLPE molecular structure and then realize the effective characterization of the aging degree and microstructure deterioration of the materials. The typical functional group transition absorption of XLPE mainly occurs in the range of 500–4000 cm^−1^. By measuring the transmittance corresponding to each characteristic wavenumber in this interval and drawing the infrared spectrum, the relative abundance of various functional groups in the material can be quantitatively or semi-quantitatively evaluated. By comparing the measured spectra with the standard chemical bond and functional group database, the chemical composition and structure information of the samples can be further determined.

**Figure 8:**
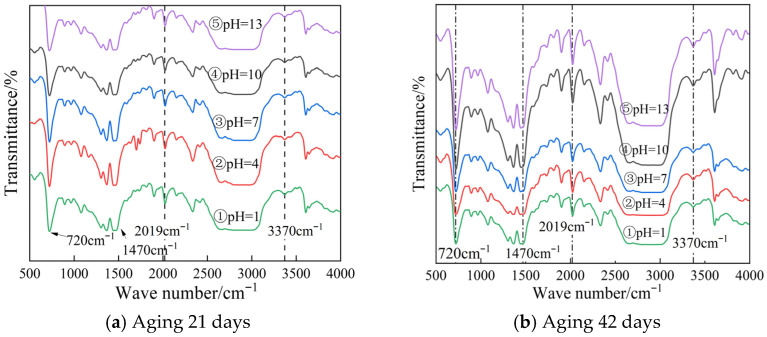
Infrared spectra of samples in different pH experimental groups.

The instrument used in this experiment is FT/IR-6100 Fourier transform infrared spectrometer from JASCO Corporation, Tokyo, Japan. The instrument wavenumber test range is 250–7800 cm^−1^, and the resolution can reach 0.5 cm^−1^. In order to meet the needs of XLPE material structure analysis, this experiment set the actual test wavenumber range of 400–4000 cm^−1^. Using the same test scheme as the above sample, the Fourier infrared spectrum test was carried out on the water tree area of XLPE cable insulation aged in different pH environments in the third group of slice samples. The test results are shown in Figure 8.

As shown in Figure 8, the stretching vibration absorption peak of free OH^−^ is usually located in the range of 3600~3200 cm^−1^. In the acidic solution, the absorption peak will shift to the low wavenumber direction, mostly in the range of 3200~3000 cm^−1^; under alkaline conditions, the OH^−^ stretching vibration absorption peak also appears in the range of 3200~3000 cm^−1^, and there is a small displacement only due to the charge effect.

Cross-linked polyethylene contains a large number of C-H bonds. When the main chain of the polymer is broken, a large amount of free methylene will be generated. Therefore, methylene can effectively reflect the changes in the internal structure of XLPE. The corresponding characteristic absorption peaks of methylene in the infrared spectrum are mainly located at 720 cm^−1^, 1470 cm^−1^, 2856 cm^−1^, and 2937 cm^−1^. By comparing the absorbance of these characteristic peaks, the evolution of the cross-linked structure in the aging area can be analyzed, and then the aging degree of the cable insulation can be characterized. However, in the actual test, the difference in sample thickness will directly affect the absorbance, and there is a certain error in the slicing process itself, so it is impossible to directly compare the absorption peak intensity in the infrared spectrum. In order to eliminate the interference of sample thickness on the test results, this paper introduces the water content index *K* as an indirect evaluation index to evaluate the aging degree of the cable. In the experiment, the combined frequency vibration peak at 2019 cm^−1^ was selected as the internal standard peak to deduct the influence of thickness on the absorption peak intensity. The formula for calculating the water content index *K* of the sample is shown in Equation (8):

(8) $$K=\frac{A_{3370}}{A_{2019}}$$

In the equation, *K* is the water index; *A*_3370_ is the absorbance of the O-H bond stretching vibration peak at the wavenumber of 3370 cm^−1^, %; and *A*_2019_ is the absorbance of the combined frequency vibration peak at the wavenumber of 2019 cm^−1^, %.

Before calculating the water content index *K*, it is necessary to convert the transmittance into absorbance. The formula is shown in (9):

(9) $$A_{i}=\mathrm{lg}\frac{1}{T_{i}}$$

In the formula, *A_i_* is absorbance, %, and *T_i_* is the transmittance, %.

Substituting the two transmittances corresponding to the O-H bond in Figure 8 into Equations (8) and (9), the water content index is calculated for all sections of each cable sample, and the average value is used as the final water content index of the sample. The water content index of the samples of different pH test groups after aging treatment is shown in Table 4.

It can be seen from Table 4 that the water content index *K* of the sample in the neutral environment is the smallest. The water content index *K* of the samples in weak acid and weak alkali environment is close, and both are higher than that in neutral environment; the water content index is higher in acidic environment.

Combined with the macroscopic dielectric properties test results, it can be seen that when there are a large number of H^+^ and OH^−^ in the solution, under the action of AC electric field, the two ions will undergo periodic reciprocating motion and continuously impact the polyethylene molecular chain. The long-term cumulative effect will cause the molecular chain to fatigue and eventually break. The continuous development of this process will lead to the continuous expansion of the amorphous region of the material, and the interface polarization and the structural damage of the amorphous region will be accompanied in the mixed branch region. Because the ionic radius and mass of H^+^ and OH^−^ are much smaller than those of Na^+^ and Cl^−^, their impact behavior on the molecular chain is more random. Macroscopically, the water branch area is larger and more dispersed, and the hydrophilic area in the dendritic aging area is also larger. Therefore, in the acid-base environment, the water content index of cable insulation after aging is higher. The changes in O–H and C–H peaks directly indicate the oxidative degradation and main chain scission of XLPE polymer, which is the fundamental reason for the deterioration of macroscopic dielectric properties.

### 4.4. Cable Aging Prediction Model Based on Macroscopic Parameters Characterization

In order to establish the prediction model of mixed tree aging growth of XLPE cable insulation in different ionic solutions, the macroscopic performance parameters of cable samples are correlated with the water tree density, so as to construct the corresponding relationship between macroscopic parameters and microscopic morphology parameters. Before the model fitting, it is necessary to calculate the mixed branch density based on the microscopic morphology observation results.

After calculation, the insulation layer volume of the cable aging area used in this test is 58,512.13 mm^3^. The total volume of the mixed branches was obtained by summing the volume of the mixed branches at all pinhole defects in each sample. The mixed branch density of the cable sample is defined as the ratio of the total volume of all mixed branches of the sample to the volume of the aging area of the cable insulation layer. The formula for calculating the density of mixed branches is as follows (10):

(10) $$\rho =\frac{\frac{4\pi }{3}\sum_{i=1}^{n}L_{1i}^{2}L_{2i}}{58512.13}=\frac{\pi \sum_{i=1}^{n}L_{1i}^{2}L_{2i}}{43884.0975}$$

In Formula (10), *L*_1*i*_ represents the maximum transverse width of the mixed branch at the ith pinhole, mm, and *L*_2*i*_ is the maximum width of the longitudinal width of the mixed branch at the ith pinhole, mm. If there are electrical tree channels outside the mixed tree branch area, the volume of the electrical tree channel should also be added. The branch density of the samples in different ion test groups is calculated as shown in Table 5.

The mixed tree density of XLPE cable samples under different pH and different aging cycles was calculated. The results show that the change of mixed tree density is significantly correlated with the pH and aging time of the solution. Under the same pH condition, the mixed branch density of the sample increases with the extension of aging time, indicating that the accumulation of aging time will continue to promote the growth and development of mixed branches in the cable insulation layer, so that the volume proportion of branch defects continues to increase. During the same aging cycle, the mixed branch density of the samples in the neutral environment (pH = 7) was always the lowest in all groups, while the branch density of the samples in the acid-base environment was significantly higher than that in the neutral group, and the branch density of the strong acid (pH = 1) and strong base (pH = 13) groups was higher than that of the weak acid (pH = 4) and weak base (pH = 10) groups. Under the same alkalinity and acidity, the branch density of the acid group was higher than that of the alkaline group. This rule shows that the presence of acid and alkali ions will greatly accelerate the formation of mixed branches, and the higher the ion concentration, the more significant the promotion effect. At the same time, the promotion effect of H^+^ on the growth of mixed branches is stronger than that of OH^−^ at the same concentration. The change rule of the above mixed branch density is highly consistent with the change trend of macroscopic dielectric performance parameters and microscopic morphology observation results.

### 4.5. The Transformation Mechanism of Electric Tree to Water Tree in Different pH Test Groups

**Figure 9:**
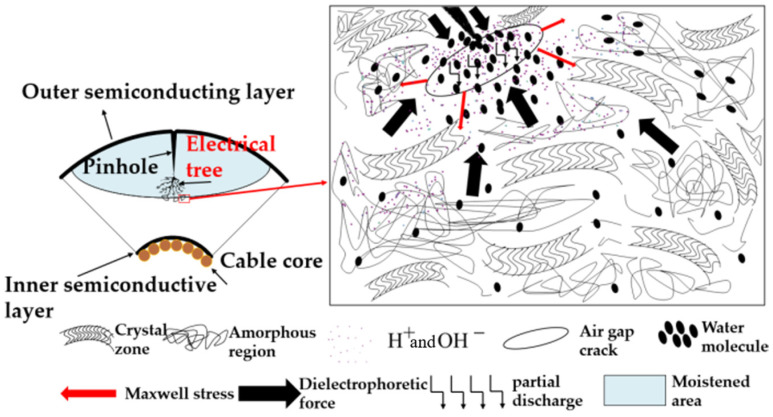
The transformation mechanism of electric tree to water tree in different pH tests.

The transformation mechanism of electrical tree to water tree in different ion test groups is shown in Figure 9. For XLPE cable samples with electrical tree defects formed inside the insulation, under the continuous action of alternating electric field, the XLPE molecular chain around the end of the electrical tree is prone to fatigue effect and microcracks. At this time, H^+^ or OH^−^ in the solution will invade the inside of the crack, greatly accelerating the diffusion rate of water in the insulating material and then forming water-filled micropores at the crack. As the water continues to penetrate into the XLPE, the area of the water-filled micropores gradually expands and aggregates. When the independent water-filled micropores continue to increase and connect, an electro-hydro mixed branch is finally formed at the end of the electrical tree.

There are significant differences in the types and concentrations of ions in different pH solutions, which in turn have a differential effect on the conversion process from electrical trees to water trees. There is a large amount of H^+^ in the acidic environment. On the one hand, it will significantly accelerate the hydrolysis reaction rate of XLPE molecular chain and strengthen the erosion effect on the end region of the electrical tree. On the other hand, H^+^ ion has small radius and high mobility, which can quickly migrate to the interior of the material under the action of external electric field, further accelerating the deterioration of the defect area. In the alkaline environment, OH^−^ is enriched, and the ion will undergo redox reaction with the methylene group on the molecular chain, change the migration law and distribution state of the conductive ion, and destroy the structural integrity of the molecular chain. Both acidic and alkaline solutions can react with XLPE molecular chains to varying degrees, which aggravates the breakage of molecular chains. The amorphous region at the end of the tree is loose, and the destruction of the molecular chain by H^+^ and OH^−^ in this region is more significant, which directly leads to a significant increase in the growth rate of the mixed branch. At the same time, the acid-base environment can also promote water molecules to participate in the redox reaction of the molecular chain more quickly, accelerate the diffusion of water inside the insulation and the formation of micropores, and ultimately promote the rapid development of mixed branches and aggravate the aging process of the cable.

As shown in Table 6, previous studies mainly focused on single electrical tree or water tree under limited environments. In contrast, this work systematically investigates electro-hydro mixed branches under five typical pH conditions and realizes multidimensional characterization from macroscopic FDS parameters to microscopic morphology. The quantitative correlation model and nonlinear parameter λ further improve the evaluation method of cable aging.

## 5. Conclusions

This work systematically studied the microstructure degradation and polymer chain fracture mechanism of XLPE under different pH environments. In order to explore the influence of solution pH on the growth characteristics of XLPE cable electro-hydraulic mixed branches, this study carried out accelerated aging tests of mixed branches under different pH environments on cable samples. By testing macroscopic dielectric performance parameters and microscopic physical and chemical parameters, the effect of pH, an environmental factor, on the aging of cable mixed branches was systematically analyzed. Combined with macro and micro double-level test methods, the comprehensive characterization and evaluation of the aging degree of cable mixed branches under different pH environments were realized. Based on the above test results and analysis, the following conclusions are drawn:

(1) The macroscopic dielectric properties test results show that the acidic and alkaline solution environment can significantly promote the accelerated aging of XLPE cable mixed branches, and the aging promotion effect is positively correlated with the concentration of H^+^ and OH^−^ in the solution. The higher the ion concentration, the more obvious the acceleration effect on the aging of mixed branches.
(2) Microscopic morphology observation and physical and chemical parameter analysis showed that the acid and alkali environment could accelerate the growth process of mixed branches. On the basis of the existing electrical trees, the strong acid and strong alkali environment was more conducive to the development of mixed branches than the weak acid and weak alkali environment, and the promotion effect of acid solution on the growth of mixed branches was more prominent than that of alkaline solution with the same concentration. This law can be reasonably explained by the difference in migration rate of different ions.
(3) Aging experiments in different acid-base environments confirmed that both acid and alkaline solutions could accelerate the growth of mixed branches to varying degrees, among which strong acid (pH = 1) and strong alkali (pH = 13) solutions had the most significant effect. Compared with Na^+^ and Cl^−^ in NaCl, H^+^, and OH^−^ have higher ion mobility and stronger ability to destroy XLPE molecular chains and polyethylene chemical bonds, which is the key reason for the rapid growth of mixed branches in acid-base environment.

## Figures and Tables

**Table 1:** Different pH solution mixed branch aging cable sample setting.

Sample No.	Electrical Tree Aging Parameters	pH Value of the Solution	Aging Parameters of Mixed Branches
pH1-1/pH1-2	12 kV, 50 Hz. Sine AC voltage Pressing time 10 min	1	7.5 kV, 400 Hz sinusoidal AC voltage Aging time aysays/42 days
pH4-1/pH4-2		4	
pH7-1/pH7-2		7	
pH10-1/pH10-2		10	
pH13-1/pH13-2		13	

**Table 2:** Nonlinear characteristic parameters of 21 days aging samples with different pH values.

Sample No.	Nonlinear Characteristic Parameter *λ*
pH1-1	2874.74
pH4-1	2543.45
pH7-1	2165.78
pH10-1	2483.83
pH13-1	2787.74

**Table 3:** Nonlinear characteristic parameters of 42 days aging samples with different pH values.

Sample No.	Nonlinear Characteristic Parameter *λ*
pH1-2	2987.73
pH4-2	2694.83
pH7-2	2898.48
pH10-2	2538.34
pH13-2	2837.83

**Table 4:** Moisture index of samples in different pH test groups.

Sample No.	Water Index *K*	Sample No.	Water Index *K*
pH1-1	0.872	pH1-2	0.889
pH4-1	0.855	pH4-2	0.863
pH7-1	0.841	pH7-2	0.856
pH10-1	0.853	pH10-2	0.861
pH13-1	0.868	pH13-2	0.879

**Table 5:** Branch density of samples in different pH test groups.

Sample No.	Branch Density/%	Sample No.	Branch Density/%
pH1-1	0.00484	pH1-2	0.00742
pH4-1	0.00442	pH4-2	0.00565
pH7-1	0.00168	pH7-2	0.00255
pH10-1	0.00435	pH10-2	0.00448
pH13-1	0.00466	pH13-2	0.00581

**Table 6:** Comparison between this work and related literature.

Literature	Research Object	Environment	Key Characterization	Main Contribution
Jiang et al. [6]	Electrical tree and water tree	Wet/dry condition	Morphology observation	Interaction between single water tree and electrical tree
Zhou et al. [11]	Electrical tree to water tree	Natural environment	Simulation and morphology	Conversion mechanism of single branch
Zhang et al. [12]	Water tree	Acidic/alkaline	Morphology	pH effect on single water tree
This article	Electro-hydro mixed branches	pH = 1, 4, 7, 10, 13	FDS + morphology + SEM + FTIR + fitting model	Systematic growth law; conversion mechanism; nonlinear parameter λ; non-destructive evaluation model

## Data Availability

The original contributions presented in this study are included in the article. Further inquiries can be directed to the corresponding author.
